# Treatment Options for Age-Related Macular Degeneration: A Budget Impact Analysis from the Perspective of the Brazilian Public Health System

**DOI:** 10.1371/journal.pone.0139556

**Published:** 2015-10-12

**Authors:** Flávia Tavares Silva Elias, Everton Nunes da Silva, Rubens Belfort, Marcus Tolentino Silva, Álvaro Nagib Atallah

**Affiliations:** 1 Oswaldo Cruz Foundation, Brasília, Distrito Federal, Brazil; 2 University of Brasilia, Brasília, Distrito Federal, Brazil; 3 Federal University of São Paulo, São Paulo, São Paulo, Brazil; 4 Federal University of Amazonas, Manaus, Amazonas, Brazil; Bascom Palmer Eye Institute, University of Miami School of Medicine, UNITED STATES

## Abstract

**Background:**

Age-related macular degeneration (AMD) is a disease that causes reduced visual acuity and blindness. The new treatment options for AMD are not provided by the Brazilian public health system.

**Objective:**

To conduct a budget impact analysis of three scenarios for the introduction of AMD treatments: all the medications (verteporfin, ranibizumab, and bevacizumab–the reference scenario), ranibizumab alone, and bevacizumab alone.

**Methods:**

The basic assumption was that the Brazilian public health system would treat the entire target population with AMD aged > 70 years between 2008 and 2011. The size of the population of interest was estimated from official population projections and the prevalence of the disease was obtained from a systematic review. Medication prices were estimated by weighting their market values with correction factors to take account of the public procurement policy. The possibility of aliquoting bevacizumab was also considered. A panel of experts was consulted to estimate the market share of the different medications for the reference scenario. The incremental costs of the ranibizumab-alone and bevacizumab-alone scenarios compared to the reference scenario were calculated. Univariate sensitivity analyses were run to check the robustness of the model.

**Results:**

In four years, the Brazilian public health system would have treated 1,136,349 individuals with AMD. The annual costs of treating one patient would have been US$476.65 for bevacizumab, US$11,469.39 for ranibizumab, and US$4,376.28 for verteporfin. The incremental cost of the ranibizumab-alone scenario would have been US$1,878,318,056.00 in four years, while the incremental cost for the bevacizumab-alone scenario would have been a reduction of US$4,978,326,359.00 (i.e., a cost saving) in the same period. The bevacizumab-alone option was found to represent a cost saving across sensitivity analyses.

**Conclusion:**

The introduction of bevacizumab for the treatment of AMD is recommended for the Brazilian Public Health System.

## Introduction

Age-related macular degeneration (AMD) is a degenerative disease that causes severe visual loss and blindness. The degenerative process affects the area of the retina called the macula, which is responsible for central visual acuity, causing distorted and blurred vision and a significant loss of visual function that compromises the activities of daily living and reduces quality of life [1,2]. The risk factors for AMD are increasing age, cigarette smoking, prior cataract surgery, and family history [3]. This disease is ranked third among the global causes of visual impairment, with a blindness prevalence of 8.70% in individuals aged > 50 years according to the World Health Organization [4].

There are two types of macular degeneration: dry (nonexudative or atrophic) and wet (neovascular or exudative). The latter is the target of the current treatments, which focus on attenuating choroidal neovascularization activity [1], hence slowing the progress of the disease. The criteria for ceasing treatment are improved visual acuity, stable lesion size, a smaller area of fluorescein leakage on angiography, and the absence of intraretinal fluid on optical coherence tomography [5].

Research has been done into pharmacological treatments for AMD. Verteporfin is used in photodynamic therapy while bevacizumab and ranibizumab are drugs that attenuate the neovascularization process when administered by intravitreal injection [5]. These drugs are not available in the Brazilian public health system.

The aim of this study was to assess the budgetary impact of three scenarios for the introduction of drugs to treat neovascular AMD by the public health system in Brazil: 1) all the medications, 2) ranibizumab alone, and 3) bevacizumab alone.

## Materials and Methods

We carried out a budget impact analysis (BIA), which followed the methodological guidelines recommended by the Brazilian Ministry of Health [6] and by MAUSKOPF et al [7]. The BIA was performed using data that reflect a specific health condition of the many elderly people. The size and characteristics of the population were calculated based on prevalence estimated for the Brazilian population. The efficacy and safety of the treatments was based on the best evidence available in the literature.

The basic assumptions were that the public health system would have provided treatment for the entire target population between 2008 and 2011, and that any one of three AMD treatment options would have been introduced in 2008: (1) availability of all three drugs (verteporfin, ranibizumab, and bevacizumab); (2) availability of ranibizumab alone, or (3) availability of bevacizumab alone. These assumptions were based on the findings of a systematic review by the *Centro Cochrane do Brasil* funded by the Ministry of Health [8].

### Target population

The number of people aged > 70 years living in Brazil between 2008 and 2011 was projected using population estimates published by the Brazilian Institute of Geography and Statistics (IBGE) [9]. We carried out a meta-analysis of AMD prevalence. The studies were selected from a systematic review [10]. The eligibility criteria were: 1) population-based; 2) random sample; 3) cross-section; 4) similar demographic features with the Brazilian population (afro-descendant, Latin American and white). We applied random effects to estimate the prevalence coefficient for two subgroups: age range of 70–79 years and age ≥80 years. To calculate the expected cases of macular degeneration, it was applied the prevalence coefficient for total population of age range of 70–79 years and the prevalence coefficient for total population of age ≥80 years. We also assumed that the neovascular form of AMD would account for two-thirds of all cases of the disease [11]. The meta-analysis was performed using STATA software V.11 (Stata Corp, College Station, TX, USA).

### Cost of medications

The drug prices were obtained from the pharmaceutical market regulatory agency (*Câmara de Regulação do Mercado de Medicamentos*) [12], which sets the maximum prices that can be charged in Brazil. A database [13] containing the amounts paid by the Brazilian public health system was used to calculate the adjustment factor based on a weighted average that computed the prices paid and the volumes acquired to model the introduction of the target medications. Bevacizumab is commercially available in 100 mg vials as a 25 mg/mL solution. The drug is neither diluted nor reconstituted. Using an aseptic technique under a laminar flow hood, a compounding pharmacist is required to aliquot approximately 0.12 mL of the solution into multiple polypropylene tuberculin or insulin syringes. Since bevacizumab can be aliquoted, we assumed [14] that 4 mL of bevacizumab would provide 31 doses, including losses, and calculated the per-dose cost for each scenario. The number of doses per year per eye treated was based on the studies carried out by the CATT Research Group [15] and by Wormald et al [16]. The treatment scheme monthly was used as baseline. The treatment scheme as-needed was considered in sensitivity analysis. Costs incurred in adverse events were not considered as the incidence of such events would not differ between the two medications [17].

### Scenarios

Considering that the Brazilian public health system did not offer any drugs for the treatment of AMD in 2008, a reference scenario was created in which all three medications would have been made available. A questionnaire given to a panel of experts who had convened to prepare guidelines for AMD at the Ministry of Health was used to estimate the respective market shares of the different treatments between 2008 and 2011. These market shares were estimated by ophthalmologists, external consultants of the Ministry of Health, and pharmacists. Based on the expert’s opinion, we calculated the average market share per year. Alongside the reference scenario, two alternative scenarios were investigated: the introduction of ranibizumab in isolation and the introduction of bevacizumab off-label in isolation. Both options were guided by the evidence available at the time [18].

### Sensitivity analysis

The incremental costs of the two alternative scenarios were calculated in comparison to the reference scenario. To evaluate the robustness of the results, a sensitivity analysis was run considering the best scenario available. The lower and upper thresholds of the following parameters were considered: prevalence (obtained from the meta-analysis of the cross-sectional studies), prices of the medications (without the adjustment factor and at a 70% discount), and aliquoting of bevacizumab: (4 mL = 1 dose [discarding the rest]) and (4 ml = 40 doses [no loss]).

## Results

**Fig 1:**
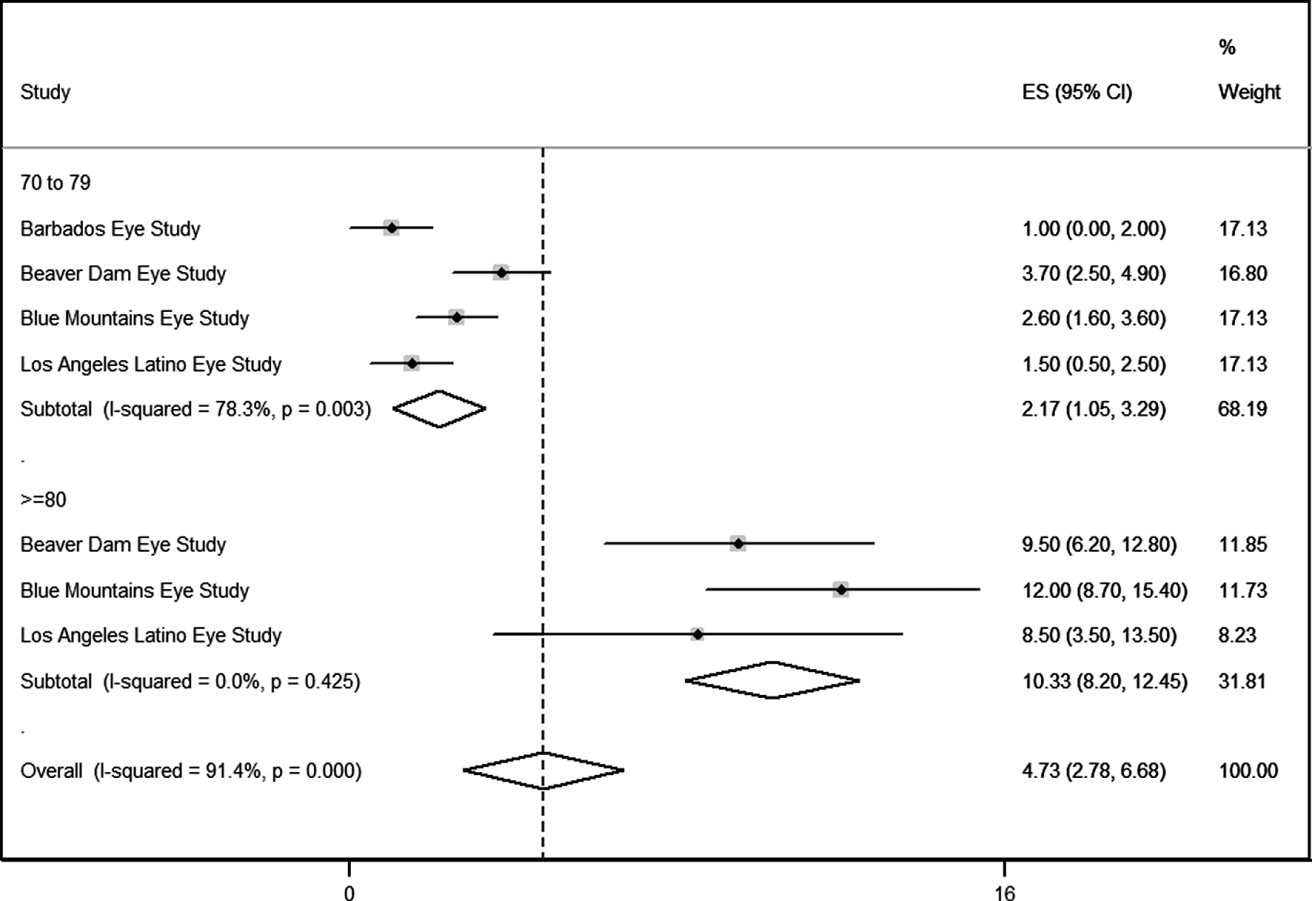
Random-effects meta-analysis of AMD prevalence.

Four studies [19 ––22] were included in the meta-analysis on AMD prevalence, which had some similarities with the Brazilian population: Latin American, Afro-descendants and white. We estimated a prevalence of 2.17 (95%CI 1.05 to 3.29) for persons aged 70–79 years and of 10.33 (95%CI 8.20 to 12.54) for persons aged≥ 80 years (Fig 1). Considering that the neovascular form of AMD would account for two-thirds of all cases of the disease, we estimated 1,136,349 individuals for the period 2008–2011 (Table 1).

**Table 1:** Estimated population with neovascular age-related macular degeneration (AMD), 2008–2011, Brazil.

Year	Age	Population ^a^	Population, AMD ^b^	Population, neovascular AMD^c^
2008	70–79	5,834,495	126,609	268,228
	≥ 80	2,669,251	275,734	
2009	70–79	6,009,549	130,407	279,292
	≥ 80	2,793,135	288,531	
2010	70–79	6,305,085	136,820	293,378
	≥ 80	2,935,585	303,246	
2011	70–79	6,350,417	137,804	295,451
	≥ 80	2,956,176	305,373	

^a^population projected,

^b^meta-analysis was conducted using random effects,

^c^ neovascular AMD would account for two-thirds of all cases of the disease.

The annual cost per patient (treatment of one eye) per drug is shown in Table 2. The cost of one year of verteporfin photodynamic therapy would have been nine times higher than the cost of treatment using bevacizumab (4 mL aliquots) while ranibizumab treatment would have cost 24 times more than bevacizumab.

**Table 2:** Annual cost of the drugs for neovascular age-related macular degeneration (2008 reference price, Brazil) (US$ million).

Drugs ^a^ ^,^ ^b^	Price (US$)	Adjustment factor^c^	Adjusted price	Per-dose cost ^d^	Doses/ year/eye treated	Cost/ year/ eye treated
Bevacizumab (4 mL)	563.88	0.83	466.69	40.05	11.9	476.65
Ranibizumab	1,723.66	0.57	980.29	980.29	11.7	11,469.39
Verteporfin (photodynamic therapy)	2,214.18	0.58	1,287.14	1,287.14	3.4	4,376.28

1 real = US$2.00.

^a^ 4 mL of bevacizumab would provide 31 doses, including losses.

^b^ Usually this options are accompanied by Intravitreal anti-VEGF injections.

^c^ The Adjustment factor refer to the weighted average of real prices paid by the health care on every purchase in the same period. This was important to set a value closer of reality.

^d^ The cost of a 4 mL vial was divided into 40 doses, then 23% was subtracted from the volume of these doses to account for losses in the aliquoting process [13]. The aliquoting process was factored in at US$25.00.

The adoption rates of the medications in the reference scenario are shown in Table 3. According to the experts consulted, if all three treatment options would have been made available, photodynamic therapy with verteporfin would have been used more in 2008 but would have been reduced over time, while the use of bevacizumab would have risen.

**Table 3:** Market share^a^ of the reference scenario, 2008–2011, Brazil.

Year	Bevacizumab (4 mL)	Ranibizumab	Verteporfin
2008	0.09	0.19	0.72
2009	0.32	0.21	0.47
2010	0.44	0.31	0.25
2011	0.59	0.34	0.07

^a^Adoption rates estimated by the expert panel.

The annual costs of the evaluated scenarios are presented in Table 4 together with the incremental costs of the ranibizumab-alone and bevacizumab-alone scenarios. The most cost-saving scenario was found to be the exclusive use of bevacizumab, which would have cost 10% of the reference scenario expenditure and would have brought savings of around US$ 5 billion over four years. By contrast, the scenario with ranibizumab treatment alone would have been the least advantageous, as it would have cost 236% more than the reference scenario, incurring a US$ 7.5 billion higher expenditure over four years.

**Table 4:** Budgetary impact (US$) of the scenarios evaluated and incremental costs (ranibizumab alone; bevacizumab alone), 2008–2011, Brazil.

Year	Reference Scenario	Costs, ranibizumab alone	Incremental cost, ranibizumab	Costs, bevacizumab alone	Incremental cost bevacizumab
2008	1,443,676,107.00	3,076,414,450.00	1,632,738,343.00	127,850,362.00	(1,315,825,745.00)
2009	1,304,484,526.00	3,203,310,999.00	1,898,826,473.00	133,123,959.00	(1,171,360,567.00)
2010	1,436,566,306.00	3,364,862,918.00	1,928,296,612.00	139,837,772.00	(1,296,728,534.00)
2011	1,335,237,773.00	3,388,648,570.00	2,053,410,796.00	140,826,261.00	(1,194,411,511.00)
Total	5,519,964,714.00	13,033,236,939.00	1,878,318,056.00	541,638,354.00	(4,978,326,359.00)

1 real = US$2.00.

The sensitivity analysis was presented in Table 5. We analyzed relevant parameters of the incremental cost of the bevacizumab-alone scenario compared with the reference scenario over the four-year study period. All the univariate analyses indicate that cost savings would have been obtained by adopting bevacizumab alone. In the worst case scenario, when the upper limit of AMD prevalence was used, a saving of US$1 billion would have been obtained. In another case scenario, with the lower limit of ranibizumab price, savings would have been US$7 billion, assuming the lowest price ceiling set for ranibizumab by the pharmaceutical market regulatory agency in Brazil. The parameter that caused the greatest variation in estimates was the prevalence of AMD, which accounted for a 34% variation. By contrast, the variable with the lowest impact on estimates was the price of bevacizumab, with a 1% variation.

**Table 5:** Sensitivity analysis of the relevant parameters of the incremental cost of the bevacizumab-alone scenarios (US$).

Parameters	Lower limit (US$) ^a^	Upper limit (US$) ^a^
Bevacizumab price	(5,060,923,102.93)	(4,951,345,962.96)
Verteporfin price	(6,298,775,461.24)	(4,091,099,059.43)
Bevacizumab fractionation	(5,007,476,993.79)	(1,091,575,112.26)
Ranibizumab price	(7,624,591,310.10)	(3,329,443,757.82)
Prevalence AMD	(6,482,922,132.09)	(1,023,454,741.24)

^a^The US$ values in the parenthesis represent negative values, it means that all parameters were cost saving.

## Discussion

### Summary of results

This study indicated that the best alternative to introducing all three existing treatments for neovascular AMD to the Brazilian public health system would have been to introduce bevacizumab alone, since it would have yielded considerable financial savings. The magnitude of the savings was around US$ 5 billion over four years. This result would have been found to be robust in the sensitivity analysis. If the Brazilian public health system had adopted this strategy, the cost of treating 1.1 million people over four years would have been approximately US$541 million.

### Validity of results

Our results should be interpreted with caution. Firstly, they derive from a model and may not express the reality of introducing pharmaceutical treatments for AMD in Brazil. For instance, one of the assumptions is that the Brazilian public health system would treat 100% of the target population—an overestimation, since it actually provides healthcare for around 75% of the general public [23]. Secondly, the prevalence of AMD among Brazilians aged > 70 years was calculated using prevalence estimates from cross-sectional studies in other countries [19–22]. There are no cross-sectional studies of the Brazilian population that could provide more accurate data. Thirdly, the adjustment factor we used to estimate the prices of the drugs purchased by the Brazilian public health system could have been underestimated. As with any product, when new drugs are introduced, prices tend to go down if the drugs are purchased in bulk by a major buyer. Finally, we assumed that the disease would affect only one eye of the patients with AMD, which would underestimate the findings. Although the symptoms normally first appear in one eye, AMD can affect both eyes [5]. This assumption was adopted because it is the most widely used method to evaluate the performance of the available drugs [24].

Despite those limitations, the sensitivity analysis suggests that the findings are consistent. At no time point was the bevacizumab-alone scenario found to be more expensive than the reference scenario, while the ranibizumab-alone scenario was the most expensive across parameters. Even when we assumed that one 4 mL vial of bevacizumab would be used to administer one 0.05 mL dose, this drug was still found to be more cost-effective. We also adapted our model to make ranibizumab alone the reference scenario if the ceiling price authorized for ranibizumab by the regulatory agency was R$733.00 (US$366.00), which would be equivalent to 21% of the price set in 2008.

We should stress that conservative parameters were used throughout the model. For instance, the adjustment factor for bevacizumab yielded a greater discount than the others. In view of this, the meta-analysis of the prevalence studies used a random model, thus increasing the amplitude of the confidence intervals. The market share of the drugs upon their entry on the market showed that bevacizumab would have a 59% share by 2011. This is comparable to the percentage reported by the American Academy of Ophthalmology, which identified from a survey which medications were the preference of 60% of its members [25].

As for the clinical performance of the different treatments for neovascular AMD, ranibizumab and bevacizumab exhibit similar effectiveness [15] and safety [17,18] [26] and are known to be more effective than verteporfin [8] [16]. Thus, the budget impact analysis was able to identify the best strategy to be adopted by the Brazilian public health care system.

### Comparison with the literature

We found only one abstract accepted in a pharmacoeconomic congress [27], in which bevacizumab was compared to ranibizumab in terms of budget impact for AMD in Brazil. The data presented in that abstract suggest that the authors did not weight neovascular AMD, thus overestimating the target population. Curiously, their analysis assumes that a 4 mL vial of bevacizumab could yield 80 doses, which is inconsistent with the available pharmaceutical formulation information [28]. Those assumptions introduced biases that precluded the generalization of the findings.

One study in the United States evaluated the economic impact on Medicare of two-year treatment for AMD [24]. The average annual cost for a patient using bevacizumab was calculated to be US$3,493.85 in the first year and US$1,852.88 in the second. The cost for patients using ranibizumab was US$16,114.52 and US$13,971.44 in the first and second years, respectively. In CATT GROUP RESEARCH [15], the annual cost of monthly treatment was calculated to be US$23,400.00 for ranibizumab (US$2,000.00/dose) and U$595.00 for bevacizumab (US$50.00/dose). A study [29], investigating different treatment regimens demonstrated that the cost per patient per eye varied between £9,656.00 (continuous treatment) and £6,398.00 (discontinuous treatment) for ranibizumab and £1,654.00 and £1,509.00 for bevacizumab, respectively. The differences between these findings and our study reflect the methods to calculate costs, especially those of services and procedures, which were not included in the analysis because the priority was to conduct an incremental cost analysis. Another American study [30] focuses on the diabetic macular edema in addition to age-related macular degeneration concluded vast savings to government-funded healthcare system by switching to bevacizumab.

### Setting

In 2008, members of the Brazilian Network for Health Technology Assessment started to examine alternative treatments for AMD [31]. Since 2011, bevacizumab has been recommended by the National Commission for Technology Incorporation into the Public Health System. The clinical guidelines for treatment were submitted to public consultation in that same year [32]. The ophthalmology centers accredited by the Ministry of Health were in agreement with the implementation of the guidelines. However, issues related to the regulation of medicines in the Brazilian market remain unresolved for lack of clearance by the Brazilian Health Surveillance Agency.

In the same time, the Brazilian government currently has stimulated preventive program for elderly people. This action is called Strategic Action Plan to Tackle Non-Communicable Chronic Diseases in Brazil [33], which is implemented at primary healthcare. Despite being outside the scope of this study, another pressing issue is the use of antioxidant and/or zinc supplements in preventive program for elderly people with AMD.

## Conclusions

Our analysis aimed to highlight that since 2008 the introduction of bevacizumab in the Brazilian public health system has been a pressing issue. Evidence of good quality have been available to the Brazilian government for eight years and no effective action has been done since then, mostly because the pharmaceutic lobby. Thus, we believe our results contributed to shed light on this problem, by bringing new evidence on cost saving in introducing bevacizumab.

The present budget impact analysis showed that the most advantageous scenario for entry of AMD treatments into the Brazilian public health system would have been to introduce bevacizumab alone rather than all the existing medications or ranibizumab alone. The costs in the bevacizumab scenario were considerably lower, yielding financial savings compared to the other scenarios. These results were corroborated by the sensitivity analysis. Given that the Brazilian public health system does not offer any effective treatment for managing neovascular AMD, we recommend the introduction of bevacizumab as the best option in this context. For future research, we recommend perform similar work investigating the budgetary impact of antioxidant and/or zinc supplements as a preventative measure for people with high risk for AMD.
